# The acid-sensing nociceptor TRPV1 controls breast cancer progression in bone via regulating HGF secretion from sensory neurons

**DOI:** 10.21203/rs.3.rs-3105966/v1

**Published:** 2023-07-05

**Authors:** TATSUO OKUI, Masahiro Hiasa, Kenji Hata, G David Roodman, Masako Nakanishi, Toshiyuki Yoneda

**Affiliations:** Shimane University Faculty of Medicine; The University of Tokushima Graduate School of Dentistry; Osaka University Graduate School of Dentistry; Indiana University; Wakayama Medical University School of Medicine; Osaka University Graduate School of Dentistry

## Abstract

Cancers showing excessive innervation of sensory neurons (SN) in their microenvironments are associated with poor outcomes due to promoted growth, increased tumor recurrence, metastasis, and cancer pain, suggesting SNs play a regulatory role in cancer aggressiveness. Using a preclinical model in which mouse 4T1 breast cancer (BC) cells were injected into the bone marrow of tibiae, we found 4T1 BC cells aggressively colonized bone with bone destruction and subsequently spread to the lung. Of note, 4T1 BC colonization induced the acidic tumor microenvironment in bone in which SNs showed increased innervation and excitation with elevated expression of the acid-sensing nociceptor transient receptor potential vanilloid-1 (TRPV1), eliciting bone pain (BP) assessed by mechanical hypersensitivity. Further, these excited SNs produced increased hepatocyte growth factor (HGF). Importantly, the administration of synthetic and natural TRPV1 antagonists and genetic deletion of TRPV1 decreased HGF production in SNs and inhibited 4T1 BC colonization in bone, pulmonary metastasis from bone, and BP induction. Our results suggest the TRPV1 of SNs promotes BC colonization in bone and lung metastasis via up-regulating HGF production in SNs. The SN TRPV1 may be a novel therapeutic target for BC growing in the acidic bone microenvironment and for BP.

## INTRODUCTION

Neurogenesis and angiogenesis are closely linked in the tumor microenvironment ^1^. It has been widely recognized that angiogenesis is essential for tumor progression and metastasis and is an established therapeutic target for cancer ^2^, while the contributions of neurogenesis to cancer progression and metastasis are poorly understood. One relevant clinical condition that suggests the importance of neurons in cancer progression is the perineural invasion (PNI). PNI, which is a pathological process frequently seen in cancers such as pancreatic, head and neck, prostate, and colorectal cancers ^3^, is histo-pathologically characterized by the invasive tumor growth in the proximity of neurons innervating the tumor microenvironment. Further, in the majority of cases, sensory neurons (SNs) around growing cancer cells show excessive sprouting, and hypersensitization and hyperexcitation, thereby inducing cancer pain ^4^. Notably, tumors with PNI are associated with high rates of post-surgical recurrence and increased metastasis, and increased cancer pain, resulting in poor outcomes ^3^. Although the mechanism of increased aggressiveness of tumors with PNI needs to be elucidated, these clinical features of PNI suggest that SNs play a role in the regulation of cancer progression.

Of note, recent studies reported that PNI is the most reliable predictive histopathological feature for the risk of bone metastasis of prostate cancer ^5^ and colorectal cancer ^6^, indicating that PNI is associated with the development and progression of bone metastasis. Breast, prostate and lung cancer has a strong predilection for spreading to bone, causing debilitating or life-limiting complications that increase morbidity and mortality ^7^. One of the most common and detrimental complications of bone metastasis is bone pain (BP) ^8,9^. Accumulating studies including ours ^10–12^ demonstrated that bone is densely innervated by SNs positive for the calcitonin gene-related peptide (CGRP^+^), the most-widely accepted selective marker for SNs ^13^. Using preclinical animal model of human ^14^ and mouse ^15^ breast cancer, and canine prostate cancer ^16^, it is reported that cancer cells injected in bone induce pathological sprouting, reorganization and excitation of CGRP^+^ SNs innervating bone, eliciting cancer-induced BP (CIBP) in association with tumor progression. These results suggest that the neurogenesis and excitation of SNs and induction of CIBP are controlled by cancer cells growing in bone. However, whether the SNs in turn regulate the tumor progression in bone as speculated in the case of PNI remains unknown.

In this study, we first determined if there are reciprocal interplays between SNs and cancer cells in CIBP and tumor progression in bone, and second, the molecular mechanism by which these interplays are controlled was investigated using a preclinical model in which the mouse 4T1 breast cancer (BC) cells were injected into the bone marrow cavity of tibiae in mice (4T1 BC mice) ^15^. The 4T1 BC cells developed the acidic tumor microenvironment in bone and progressively induced BP (BC-induced BP, BCIBP) with increased expression and activation of the acid-sensing nociceptor, transient receptor potential vanilloid-1 (TRPV1) on SNs. Further, our data demonstrate that activated TRPV1 promotes 4T1 BC progression in tibiae and pulmonary metastasis through up-regulation of the production of hepatocyte growth factor (HGF) in SNs. We propose that the SN TRPV1 controls not only BCIBP but also BC progression in bone and lung metastasis from bone.

## RESULTS

### Increased neurogenesis of SNs in tibiae colonized by 4T1 BC cells

**Figure 1:**
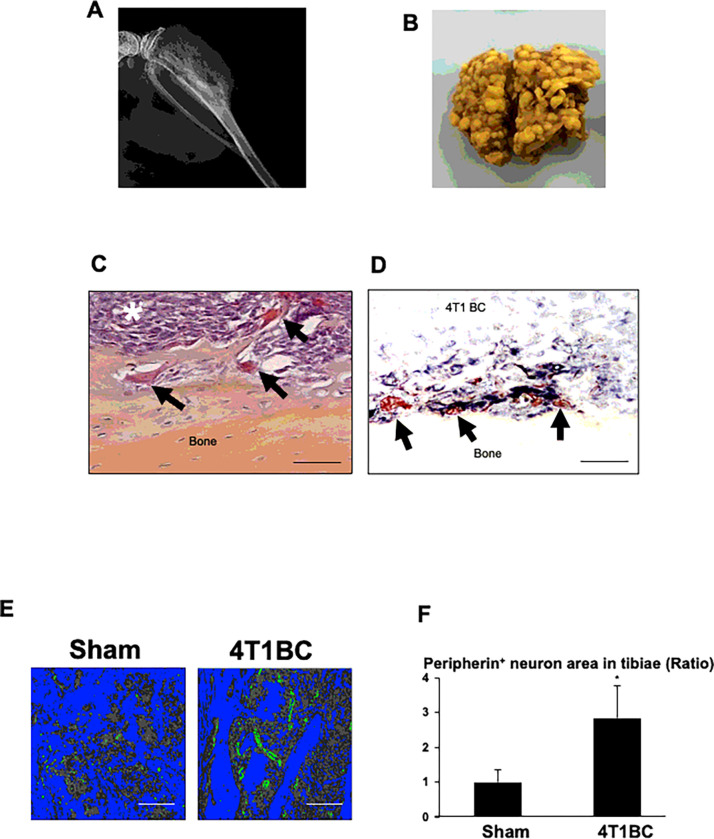
Increased innervation of SNs associated with 4T1 BC colonization in tibiae. **A.** Radiograph of osteolytic lesions associated with 4T1 BC colonization in tibiae taken at day 21 prior to sacrifice under general anesthesia. **B.** Macroscopic view of lung metastasis of 4T1 BC in the same mice as 1A. **C.** Histology of tibiae colonized by 4T1 BC cells (white asterisk). Bone-resorbing osteoclasts are seen at the endosteal surface of tibiae (black arrows). Tibiae were harvested at day 21 after sacrifice. Scale bar=100μm. **D.** Spatial interactions of peripherin^+^ SNs (black) with 4T1 BC cells and osteoclasts (red) in tibiae colonized by 4T1 BC. Sections were incubated with a primary anti-peripherin antibody (1:1,000) overnight at 4°C and then streptavidin-biotin complex, EnVision horse radish peroxidase (HRP) for 60 min and visualized using a nickel 3,3-diaminobenzidine (DAB) substrate-chromogen solution, and subsequently double-stained with tartrate-resistant acid phosphatase (TRAP) for osteoclasts using a TRACP & ALP double-stain Kit (Takara Bio, and analyzed using Magnafire 4.1 software with the IX70 microscope (Olympus). Scale bar=100μm. **E.** Peripherin^+^ SNs (green) in tibiae injected with or without (sham) 4T1 BC cells. Tibiae were harvested at day 21 and sections were immunofluorostained with a primary anti-peripherin antibody as described in Figure 1D and counterstained with DAPI. Bones and bone marrow are seen in blue and grey by image binarization using Image J, respectively. Scale bar=200μm. **F.** Quantitative analysis of Figure 1E. Peripherin^+^ area in the whole histological section of tibiae of sham and the 4T1 mice was determined under a fluorescent microscope using Neuron J. Three histological sections randomly selected were used for quantitation. Ratio represents the area of peripherin^+^ SNs in tibiae of 4T1 BC mice/the area of peripherin^+^ SNs in tibiae of sham mice x 1. Data are shown as mean ± SD (N=3). *p<0.05 *vs* sham mice.

As we previously reported ^15^, the mouse 4T1 BC cells inoculated in tibiae progressively colonized bone accompanying with osteolytic lesions (Fig. 1A). Of note, the 4T1 BC mice showed numerous tumor foci in lung (Fig. 1B), demonstrating that 4T1 BC cells colonized bone subsequently metastasized to lung. BC patients with bone metastases often develop secondary metastasis to distant visceral organs, further worsening outcomes ^17–19^.

Histological examination demonstrated that 4T1 BC cells (Fig. 1C, white asterisk) occupy the bone marrow cavity of tibiae with increased osteoclastic bone destruction (Fig. 1C, black arrows).

Immunohistochemistry (IHC) using an antibody for peripherin, one of the molecular markers for SNs ^20^, together with tartrate-resistant acid phosphatase (TRAP) staining, revealed that the peripherin^+^ SNs (Fig. 1D, black) innervate within the 4T1 tumor in close proximity of TRAP^+^ osteoclasts (Fig. 1D, black arrows), suggesting that SNs and osteoclasts establish spatial and functional interactions. Further, immunofluorescent staining showed that the innervation of the peripherin^+^ SNs was significantly increased in tibiae colonized by 4T1 BC cells compared to tibiae of sham mice (Fig. 1E and 1F). These results demonstrate that 4T1 BC colonization increased SN innervation in tibiae, developing the PNI associated with BC colonization in bone.

### SN axogenesis in the acidic microenvironment of 4T1 BC in bone

We next determined the mechanism underlying increased SN innervation in the 4T1 BC tumor in tibiae. It is reported that aggressive cancer progression is associated with the generation of acidic microenvironments ^21,22^ that facilitates growth, invasion and metastasis of cancer cells and protects them from host immune surveillance. We previously showed that the acidic bone microenvironment of multiple myeloma promotes SN innervation and excitation, inducing bone pain ^11^. We therefore determined if the 4T1 BC microenvironment in tibiae is acidic using the pH sensor fluorescent dye, acridine orange ^11^. Acridine orange staining demonstrated that the 4T1 BC microenvironment in tibiae was acidic (Supplementary Fig. 1A). Consistent with the *in vivo* results, the extracellular pH of 4T1 BC cells in culture was also acidic (Supplementary Fig. 1B), which was reversed by the treatment with bafilomycin A1, a selective inhibitor of membranous vacuolar proton pump (H^+^-V-ATPase) ^11^. We previously reported that bafilomycin A1 blocked the development of the acidic microenvironment associated with multiple myeloma in mice ^11^. Immunofluorescent staining confirmed that 4T1 BC cells expressed H^+^-V-ATPase (Supplementary Fig. 1C). We also detected the expression of the H^+^-V-ATPase in mouse BC cell line E0771 (Supplementary Fig. 1C). Taken together, these results suggest that the H^+^-V-ATPase expressed on 4T1 BC cells contributes to the generation of the acidic microenvironment in bone through secreting protons.

We then determined if the 4T1 BC acidic microenvironment promotes SN innervation using the compartmentalized microfluidic platforms in which 4T1 BC cells and the dorsal root ganglia (DRG) SN cell line F11 ^23^ were co-cultured. DRG is the cell body of primary SN fibers that transduces peripheral neural activity to electrical signal and relays to the central nervous system (CNS) ^12^. The F11 cells extended their axons toward 4T1 BC cells (Supplementary Fig. 1D and 1E). Further, axogenesis of primary rat DRG SN cells was also significantly promoted in the co-cultures with 4T1 BC cells in the lower chambers of trans-wells (Supplementary Fig. 1F and 1G). Importantly, bafilomycin A1 decreased the axogenesis of primary rat DRG SN cells in the presence of 4T1 BC cells (Supplementary Fig. 1F and 1G). These results suggest that the acidic microenvironment developed following proton release via the H^+^-V-ATPase of 4T1 BC cells promotes axonal outgrowth of SNs.

### Induction of SN excitation and BCIBP in the 4T1 BC mice

**Figure 2:**
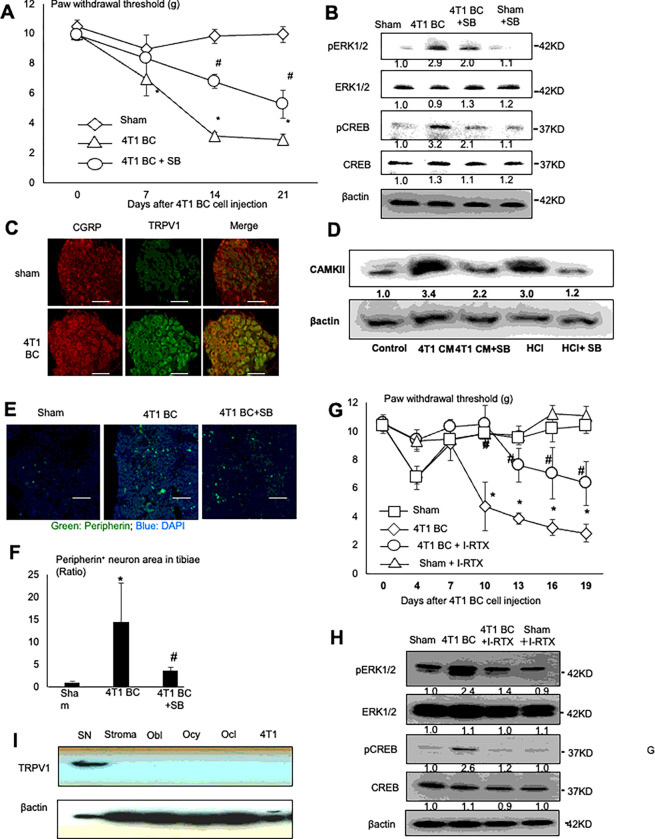
Role of TRPV1 in the sprouting and excitation of SNs, and the induction of BCIBP in the 4T1 BC mice **A.** Progression of BCIBP assessed by the hind-paw mechanical allodynia by the von Frey test in tibiae of sham and the 4T1 BC mice treated with or without a selective synthetic TRPV1 antagonist, SB366791 (500 μg/kg, ip, once a day from day 6 to 20). The force by which mice withdraw their hind-paw is shown on Y-axis as paw withdrawal thresholds (g) in the figure. Mice with BCIBP in tibiae show decreased paw withdrawal threshold. Data are shown as mean ± SD (N=5). *p<0.05 *vs* sham mice. ^#^p<0.05 *vs* untreated 4T1 BC mice. **B.** Expression of pERK1/2 and pCREB, two molecular markers for SN excitation, in lumbar DRGs of sham and the 4T1 BC mice treated with or without SB366791. Lumbar (L3-L5) DRGs were harvested from mice of each treatment group after sacrifice at day 21 and subjected to Western analysis. **C.** Co-expression of CGRP **(left)** and TRPV1 **(center)** on DRG SNs of the 4T1 BC mice. DRGs were harvested from sham and the 4T1 BC mice after sacrifice at day 21 and sections were immunofluorostained with a primary anti-CGRP (1:200) and anti-TRPV1 (1:1,000) antibody overnight at 4 C, and a secondary fluorescent-labeled antibody (1:100) for 60 min. Scale bar=50μm. **D.** Activation of TRPV1 signal pathway by 4T1 BC CM. Rat immortalized DRG SN cell line 50B11 was treated with 4T1 BC CM (30%, v/v), which is acidic (see Supplemental Figure 1B), or hydrochloric acid (HCl, pH 6.5) as positive control in the absence or presence of SB366791 (200 nM) for 30 min, lysed and subjected to Western analysis for the expression of Ca^2+^/calmodulin-dependent protein kinase II (CAMKII). **E.** Increased peripherin^+^ SNs (green) in tibiae of the 4T1 BC mice treated with or without SB366791. Tibiae were harvested from mice after sacrifice at day 21 and sections were immunofluorostained with a primary anti-peripherin antibody (1:1,000) and counterstained with DAPI (blue). Scale bar=200μm. **F.** Quantitative analysis of Figure 2E. Peripherin^+^ area in the whole histological section of tibiae of sham and the 4T1 mice was determined under a fluorescent microscope using Neuron J. Three histological sections randomly selected were used for quantitation. Ratio represents peripherin^+^ SN of 4T1 BC or 4T1 BC + SB366791/peripherin^+^ SN of sham mice x 1. Data are shown as mean ± SD (N=3). *p<0.05 *vs* sham mice. ^#^p<0.05 *vs* untreated 4T1 BC mice. **G.** Effects of a selective natural antagonist of TRPV1, I-RTX (0.5 μmol/kg, ip, once a day from day 6 to 20) on BCIBP in the 4T1 BC mice. Mechanical allodynia of sham mice seen at day 4 was due to surgical trauma. Data are shown as mean ± SD (N=5). *p<0.05 *vs* sham mice. ^#^p<0.05 *vs* untreated 4T1 BC mice. **H.** Effects of I-RTX on the expression of pERK1/2 and pCREB in DRGs of the 4T1 BC mice. DRGs were harvested from mice of each treatment group after sacrifice at day 21 and subjected to Western analysis. **I.** Western analysis of TRPV1 expression in DRGs of 4T1 BC mice, and mouse primary bone marrow stromal cells, primary calvarial osteoblasts (Obl), MLO-Y4 osteocyte-like cells (Ocy) and osteoclasts generated from bone marrow macrophages (Ocl) and 4T1 BC cells.

Protons are a potent pain-inducing substance ^24^ and cancers with PNI are frequently associated with pain ^3^. We therefore examined if the 4T1 BC mice, in which PNI and acidic microenvironment developed in tibiae, show BCIBP by assessing the hind-paw mechanical allodynia using the von Frey test. The 4T1 BC mice demonstrated progressive BCIBP in association with 4T1 BC tumor growth (Fig. 2A). Further, the lumbar DRGs (L3-L5) that innervate hind-limb ^25^ showed an up-regulation of the phosphorylation of the extracellular receptor kinase 1/2 (pERK1/2) and cyclic AMP-responsive element-binding protein (pCREB), which are widely-accepted molecular indicators for SN excitation ^26^ by Western analysis in the 4T1 BC mice compared to sham mice (Fig. 2B). These results show that SN excitation and BCIBP are induced in the tibiae of the 4T1 mice.

### Role of TRPV1 in SN excitation and BCIBP induction in the 4T1 BC mice

Acid has been known to induce pain via activation of the acid-sensing nociceptors expressed on SNs such as TRPV1 and acid-sensing ion channels ^24^. TRPV1, a Ca^2+^-permeable non-selective cation channel, regulates excitation, sensitization and function of SNs, leading to the induction of pain ^27^. We reported that SN excitation and bone pain associated with lung cancer are decreased in TRPV1^−/−^ mice ^28^. Therefore, we determined if TRPV1 plays a role in the induction of SN excitation and BCIBP in the 4T1 BC mice. Immunohistochemical examination using the anti-TRPV1 and anti-CGRP antibody showed that TRPV1 expression on CGRP^+^ SNs in the DRGs (L3) is increased in the 4T1 BC mice compared to sham mice (Fig. 2C). These results together with Fig. 2A and 2B suggest that increased TRPV1 expression on SNs in tibiae stimulates SN excitation and BCIBP induction.

We verified that TRPV1 on SNs is activated by the 4T1 BC acidic microenvironment by showing increased expression of Ca^2+^/calmodulin-dependent protein kinase II (CAMKII), a TRPV1 downstream signal molecule ^29^, in the 50B11 rat immortalized DRG SN cell line ^30^ that was treated with acidic 4T1 BC CM or hydrochloric acid (pH 6.5) (Fig. 2D). Importantly, SB366791, a selective synthetic antagonist for TRPV1 ^31^, decreased CAMKII expression that was up-regulated by acidic 4T1 BC CM and hydrochloric acid.

We then determined the role of TRPV1 activation in the promotion of SN neurogenesis and induction of BCIBP in the 4T1 BC mice. Treatment of the 4T1 mice with SB366791 significantly decreased hind-paw mechanical allodynia (Fig. 2A) and pERK1/2 and pCREB expression in DRGs of the 4T1 BC mice (Fig. 2B). These results suggest that TRPV1 activation is necessary for the promotion of SN excitation and induction of BCIBP. Further, increased number of peripherin^+^ SNs in tibiae in the 4T1 mice was significantly reduced by the treatment with SB366791 (Fig. 2E and 2F), validating that TRPV1 activation promotes SN neurogenesis in tibiae.

In addition, 5-iodoresiniferatoxin (I-RTX), a selective natural antagonist of TRPV1 ^32^ also reduced BCIBP (Fig. 2G) and down-regulated pERK1/2 and pCREB expression in DRGs of 4T1 BC mice (Fig. 2H). These results demonstrate that both synthetic (SB366791) and natural (I-RTX) TRPV1 antagonists alleviate SN excitation and BCIBP induction in the 4T1 BC mice, confirming that TRPV1 activation is essential for the promotion of SN excitation and induction of BCIBP.

Earlier studies described that TRPV1 is expressed in cancers including BC ^33^, and osteoblasts and osteoclasts ^34^, raising the argument that the TRPV1 expressed on non-neuronal cells residing in the 4T1 BC microenvironment may play a role in the axogenesis and excitation of SNs and induction of BCIBP as well. We therefore determined relative TRPV1 expression in the lumbar DRGs harvested from the 4T1 BC mice, compared with that in 4T1 BC cells, and primary mouse bone marrow stromal cells, and osteoclasts formed from mouse bone marrow macrophages, and mouse primary calvarial osteoblasts, and mouse MLO-Y4 osteocyte-like cells. Western analysis showed that TRPV1 is intensely expressed in DRGs, while little expression was detected in other cells examined (Fig. 2I). These results show that TRPV1 is predominantly expressed in DRG SNs as reported ^35^ in the acidic 4T1 BC microenvironment, and suggest that SN TRPV1 plays a primary role in SN excitation and neurogenesis, and BCIBP induction.

### Role of TRPV1 in angiogenesis in the 4T1 BC mice

Since neuron remodeling has been known to coordinate with angiogenesis ^1^, we determined the role of TRPV1 in angiogenesis associated with 4T1 BC colonization in tibiae. IHC showed that CD31^+^ blood vessel formation in tibiae is increased in the 4T1 BC mice (Supplementary Fig. 2A and 2B), which was significantly decreased by SB366791 treatment. (Supplementary Fig. 2A and 2B). These results suggest that TRPV1 activation in the acidic 4T1 BC microenvironment promotes tumor angiogenesis as well as neurogenesis.

### Role of TRPV1 in 4T1 BC progression in tibiae and metastasis to lung from tibiae

**Figure 3:**
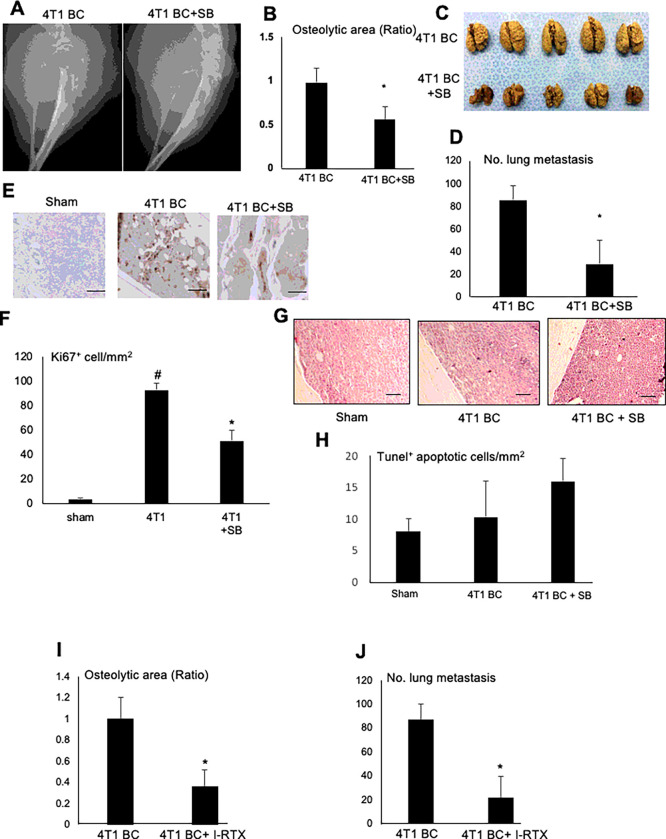
Effects of TRPV1 antagonists, SB366791 (synthetic) and I-RTX (natural), on 4T1 BC colonization in tibiae, metastasis to lung, and weight loss in the 4T1 BC mice. **A.** Radiograph of osteolytic lesions associated with 4T1 BC colonization in tibiae in the 4T1 BC mice treated without **(left)** or with **(right)** SB366791 (500 μg/kg, ip, once a day from day 6 to 20) taken at day 21 prior to sacrifice under general anesthesia. **B.** Quantitative analysis of Figure 3A. Ratio represents osteolytic area of 4T1 BC + SB366791/osteolytic area of 4T1 BC x 1. Data are shown as mean ± SD (N=5). *p<0.05 *vs* 4T1 BC mice. **C.** Macroscopic view of lung metastasis of 4T1 BC from tibiae in the same experiment as Figure 3A. **D.** Quantitative analysis of Figure 3C. Lungs were harvested from mice after sacrifice and fixed in Bouin’s fixative, and the number of metastatic foci were macroscopically counted. Data are shown as mean ± SD (N=5). *p<0.05 *vs* 4T1 BC mice. **E.** Effects of SB366791 (500 mg/kg, ip, once a day from day 6 to 20) on 4T1 BC cell proliferation in bone evaluated by Ki-67 expression. Bones were harvested at day 21 and sections were immunostained with anti-Ki67 antibody. Scale bar=200μm. **F.** Quantitative analysis of Figure 3E. Ki-67^+^ 4T1 BC cells in three fields of 1 mm^2^ of tumor were counted under a microscopy using Image J. Data are shown as mean ± SD (N=5). *p<0.05 *vs* sham mice. ^#^p<0.05 *vs* untreated 4T1 BC mice. **G.** Effects of SB366791 (500 mg/kg, ip, once a day from day 6 to 20) on 4T1 BC cell apoptosis in tibiae of 4T1 BC mice. Tibiae were harvested at day 21 and sections were stained with TUNEL. Apoptotic 4T1 BC cells are seen in brown. Scale bar 200μm. **H.** Quantitative analysis of Figure 3G. The number of TUNEL-positive 4T1 BC cells in three fields of 1 mm^2^ of tumor were counted under a microscopy using Image J. Data are shown as mean ± SD (N=5). *p<0.05 *vs* sham and untreated 4T1 BC mice. **I.** Osteolytic lesions associated with 4T1 BC colonization in tibiae in 4T1 BC mice treated with or without I-RTX (0.5 μmol/kg, ip, once a day from day 6 to 20). Ratio represents osteolytic area of 4T1 BC + I-RTX/osteolytic area of 4T1 BC x 1. Data are shown as mean ± SD (N=5). *p<0.05 *vs* 4T1 BC mice. **J.** Lung metastasis of 4T1 BC from tibiae in the same experiment as Figure 3I. Data are shown as mean ± SD (N=5). *p<0.05 *vs* 4T1 BC mice.

While we investigated the role of TRPV1 in SN neurogenesis and excitation and BCIBP induction, we found that the 4T1 BC progression in tibiae (Fig. 3A and 3B) and metastasis to lung from tibiae (Fig. 3C and 3D) were significantly reduced in the 4T1 BC mice treated with SB366791. SB366791 also significantly decreased 4T1 BC cell proliferation as shown by Ki67 staining (Fig. 3E and 3F). SB366791 increased apoptosis in 4T1 BC cells as shown by TUNEL staining in tibiae (Fig. 3G and 3H), although the results are not significantly different.

Further, I-RTX also decreased 4T1 BC progression in tibiae (Fig. 3I) and lung metastasis from tibiae (Fig. 3J). These results suggest that TRPV1 plays an important role not only in SN axogenesis and excitation and BCIBP induction but also 4T1 BC progression in tibiae and lung metastasis from tibiae.

Of interest, SB366791 failed to decrease 4T1 BC growth at the mammary fat pad (Supplementary Fig. 3A). Although the reason for this is unclear, the differences in the tumor microenvironment, subtype of innervating SN, and the level of TRPV1 expression and activation between bone and mammary fat pad may account for this failure. Whatever the mechanism, the results suggest that bone may be one of the preferential target sites for TRPV1 inhibition to suppress BC progression.

Another notable observation in these experiments was that the 4T1 BC mice showed considerable weight loss (Supplementary Fig. 3B) despite mice were pair-fed. Treatment with SB366791 (Supplementary Fig. 3B) or I-RTX (Supplementary Fig. 3C) significantly prevented the weight loss. Since weight loss is one of the characteristics of cancer cachexia ^36^, these results suggest that blockade of TRPV1 activation may prevent the development of cachexia, thereby prolonging survival. Related to our results, TRPV1^−/−^ mice were shown to have longer life-span than WT mice ^37^.

### BC progression in tibiae and metastasis to lung from tibiae in TRPV1^−/−^ mice

**Figure 4:**
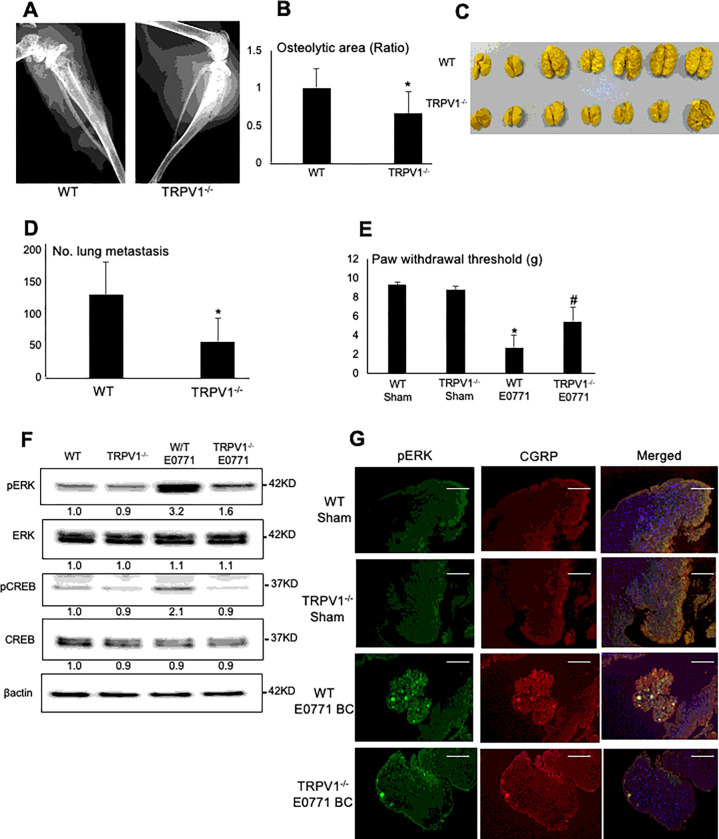
Colonization in tibiae and lung metastasis of E0771 BC cells from tibiae, and BCIBP induction in TRPV1^−/−^ mice. **A.** Radiograph of osteolytic lesions associated with mouse E0771 BC colonization in tibiae in WT **(left)** and TRPV1^−/−^ **(right)** mice taken at day 21 prior to sacrifice under general anesthesia. **B.** Quantitative analysis of Figure 4A. Ratio represents osteolytic area of TRPV1^−/−^ mice /osteolytic area of WT mice x 1. Data are shown as mean ± SD (N=7). *p<0.05 *vs* WT mice. **C.** Macroscopic view of lung metastasis of E0771 BC cells from tibiae in the same experiment as Figure 4A. **D.** Quantitative analysis of Figure 4C. The number of 4T1 BC metastatic foci in lung was macroscopically counted as described in Figure 3D. Data are shown as mean ± SD (N=7). *p<0.05 *vs* WT mice. **E.** Hind-paw mechanical allodynia assessed at day 21 in WT and TRPV1^−/−^ mice that were intratibially injected with E0771 BC cells. See Figure 2A legend for experimental details. Data are shown as mean ± SD (N=7). *p<0.05 *vs* sham WT mice. ^#^p<0.05 *vs* WT E0771 mice. **F.** Expression of molecular marker for SN excitation, pERK1/2 and pCREB, in DRGs harvested from WT and TRPV1^−/−^ mice that were intratibially injected with E0771 BC cells at day 21 by Western analysis. **G.** Co-expression of pERK1/2 **(left)** and CGRP **(center)** in SNs in DRGs harvested from WT andTRPV1^−/−^ mice intratibially injected with or without E0771 at day 21 by immunofluorescence. Scale bar=100μm.

To further investigate the importance of TRPV1 in BC, we determined BC progression in tibiae and lung metastasis from tibiae in TRPV1^−/−^ mice compared with wild-type (WT) mice. Here we employed the mouse E0771 BC cells ^38^. Similar to 4T1 BC cells, E0771 BC cells intratibially injected in C57BL/6 WT mice colonized tibiae with multiple osteolytic lesions (Fig. 4A and 4B) and showed metastases to lung (Fig. 4C and 4D). In contrast, E0771 BC colonization in tibiae (Fig. 4A and 4B) and lung metastasis (Fig. 4C and 4D) were significantly decreased in TRPV1^−/−^ mice. These results confirm a critical role in the promotion of BC progression in tibiae and lung metastasis from tibiae.

### Induction of BCIBP in TRPV1^−/−^ mice

We also determined BCIBP induction in E0771 BC-injected TRPV1^−/−^ mice compared to E0771 BC-injected WT mice. E0771 BC-injected TRPV1^−/−^ mice exhibited significantly decreased mechanical allodynia (Fig. 4E) and reduced expression of pERK1/2 and pCREB in DRGs (Fig. 4F) compared to WT mice, indicating that BCIBP and SN excitation are also regulated by TRPV1. Immunofluorescence staining of DRGs showed increased pERK1/2 expression on CGRP^+^ SNs in WT mice (Fig. 4G), while pERK1/2 expression on CGRP^+^ SNs was decreased in DRGs in TRPV1^−/−^ mice, suggesting that the excitation of SNs in DRGs is regulated by TRPV1 (Fig. 4G). These results verify that TRPV1 contributes to the induction of BCIBP in E0771 BC-bearing mice.

### Expression of HGF in SNs in DRG and bone

**Figure 5:**
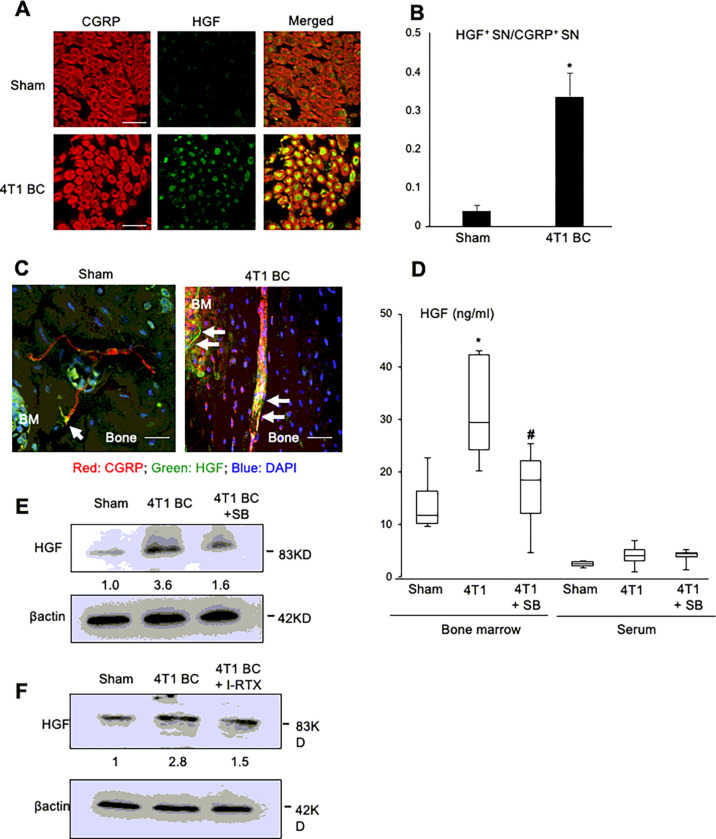
HGF expression in SNs in tibiae and DRG. **A.** Co-expression of CGRP **(left, red)** and HGF **(center, green)** on SNs in DRGs of sham **(top)** and 4T1 BC **(bottom)** mice. DRGs were harvested from mice at day 21 and sections were immunofluorostained with anti-CGRP and anti-HGF antibody. Scale bar=50μm. **B.** Quantitative analysis of Figure 5A. The number of CGRP^+^ and HGF^+^ SNs was enumerated under a fluorescent microscope using Neuron J software. The value on Y-axis shows the number of HGF^+^ SNs/the number of CGRP^+^ SNs. Data are shown as mean ± SD (N=3). *p<0.01 *vs* sham mice. **C.** Co-expression of HGF on CGRP^+^ SNs (white arrows) in the cortical bone of tibiae of sham (left) and 4T1 BC mice (right). Tibiae were harvested from mice at day 21 and sections were immunofluorostained with anti-CGRP (red) and anti-HGF (green) antibody and counterstained with DAPI (blue). Scale bar=50μm. **D.** HGF levels in bone marrow and serum of sham, untreated 4T1 BC mice and 4T1 BC mice treated with SB366791 (500μg/kg, ip, once a day from day 6 to 20) by ELISA (R & D Systems, #MHG00, Mouse/Rat HGF Quantikine ELISA Kit). Bone marrow and blood were harvested at day 21 prior to sacrifice. Data are shown as mean ± SD (N=8). *p<0.01 *vs* sham mice. ^#^p<0.05 *vs* untreated 4T1 BC mice. **E.** Western analysis of HGF expression in DRGs harvested from sham, untreated 4T1 BC mice and 4T1 BC mice treated with or without SB366791 (500μg/kg, ip, once a day from day 6 to 20) at day 21. **F.** Western analysis of HGF expression in DRGs of the 4T1 BC mice treated with or without I-RTX (0.5 μmol/kg, ip, once a day from day 6 to 20). DRGs were harvested at day 21.

We next determined the mechanism of the regulation of osteolytic cancer cells progression in tibiae and lung metastasis from tibiae following TRPV1 activation. It has been shown that neurons communicate with surrounding tumors by releasing soluble tumor growth-stimulating factors in a paracrine manner in PNI ^39^. We therefore investigated if SNs produce a tumor growth-stimulating factor for osteolytic cancer cells. To approach this, we conducted microarray analysis between the rat primary DRGs tibial injected with and without osteolytic cancer cells IP-B12 ^40^. Of interest, microarray analysis showed that the expression of HGF, a potent growth-stimulating factor for cancer cells ^41^, is substantially up-regulated in the DRGs tibial injected with IP-B12 (Supplementary Fig. 4A). Consistent with the microarray analysis, IHC using anti-CGRP and anti-HGF antibody showed increased HGF expression in CGRP^+^ DRG SNs in the 4T1 BC mice compared to sham mice (Fig. 5A and 5B) and intense HGF expression on CGRP^+^ SNs running through the cortical bone in the 4T1 BC mice (Fig. 5C, right, white arrows). In contrast, HGF expression on CGRP^+^ SNs in bone in sham mice is relatively low (Fig. 5C, left, white arrow).

As can be seen in Fig. 5C, the bone marrow also showed strong HGF expression. It has been shown that the bone marrow stromal cells produce HGF to support cancer colonization in bone ^42^. Western analysis show that the bone marrow stromal cells produce HGF to the comparable level to that of DRG SN cells (Supplementary Fig. 4B, see Control lane in stromal cells and DRG SNs). However, while HGF production by DRG SN cells was increased when co-cultured with 4T1 BC cells, bone marrow stromal cells, which express little TRPV1 (Fig. 2I), did not increase HGF production in the co-cultures with 4T1 BC cells (Supplementary Fig. 4B). These results suggest that SNs are primarily responsible for increased HGF expression in the acidic 4T1 BC tumor microenvironment in tibiae. However, the contribution of the bone marrow stroma-derived HGF is not excluded.

ELISA (R & D Systems, #MHG00, Mouse/Rat HGF Quantikine ELISA Kit) showed that HGF levels in the bone marrow of tibiae of the 4T1 mice were significantly elevated (Fig. 5D), whereas circulating serum levels of HGF were not changed in the 4T1 mice. Western analysis also demonstrated increased HGF expression in the DRG SNs of the 4T1 BC mice (Fig. 5E). These results suggest that the effect of 4T1 BC colonization in bone on HGF production is local and restricted to the bone marrow cavity.

Importantly, ELISA demonstrated that HGF production in the bone marrow in the 4T1 BC mice were decreased by the treatment with SB366791 (Fig. 5D). Similarly, Western analysis showed that HGF production in DRG SNs was decreased by the treatment with SB366791 (Fig. 5E) or I-RTX (Fig. 5F).

**Figure 6:**
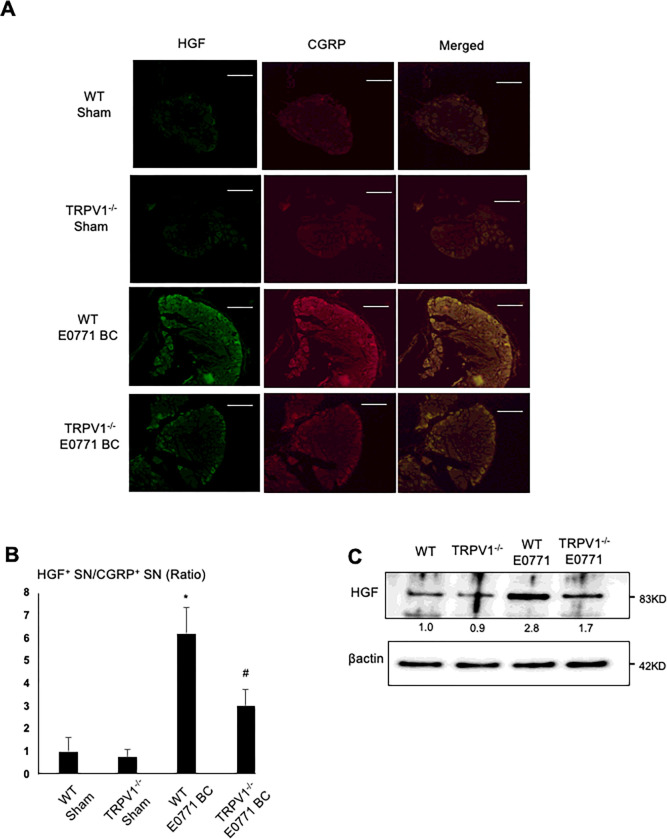
HGF expression on CGRP^+^ SNs **A.** Co-expression of HGF **(left)** and CGRP **(center)** on SNs in DRGs in WT and TRPV1^−/−^ mice intratibially injected with or without E0771 BC cells. DRGs were harvested from mice at day 21 and sections were immunofluorostained with anti-CGRP and anti-HGF antibody. Scale bar=50μm. **B.** Quantitative analysis of Figure 6A. Ratio represents TRPV1^−/−^ sham, E0771 BC-injected WT or E0771 BC-injected TRPV1^−/−^ mice /WT sham mice x 1. Data are shown as mean ± SD (N=7). *p<0.05 *vs* WT sham mice. ^#^p<0.05 *vs* WT E0771 BC mice. **C.** Western analysis of HGF expression in DRG SNs harvested at day 21 from WT and TRPV1^−/−^ mice that were intratibially injected with or without E0771 BC cells.

Consistent with these results, IHC of DRGs revealed that the up-regulation of HGF expression on CGRP^+^ SNs in DRGs in the presence of E0771 BC in bone is significantly less in TRPV1^−/−^ mice than WT mice (Fig. 6A and 6B). Western analysis also showed that the increase in HGF expression in the presence of E0771 BC cells is less in DRGs of TRPV1^−/−^ mice than WT mice (Fig. 6C). Taken together, these results indicate that TRPV1 plays an important role in the regulation of HGF expression in SNs in DRGs and bone.

### Role of HGF derived from SNs in the 4T1 BC colonization in tibiae, and lung metastasis from tibiae, and induction of BCIBP in the 4T1 BC mice

**Figure 7:**
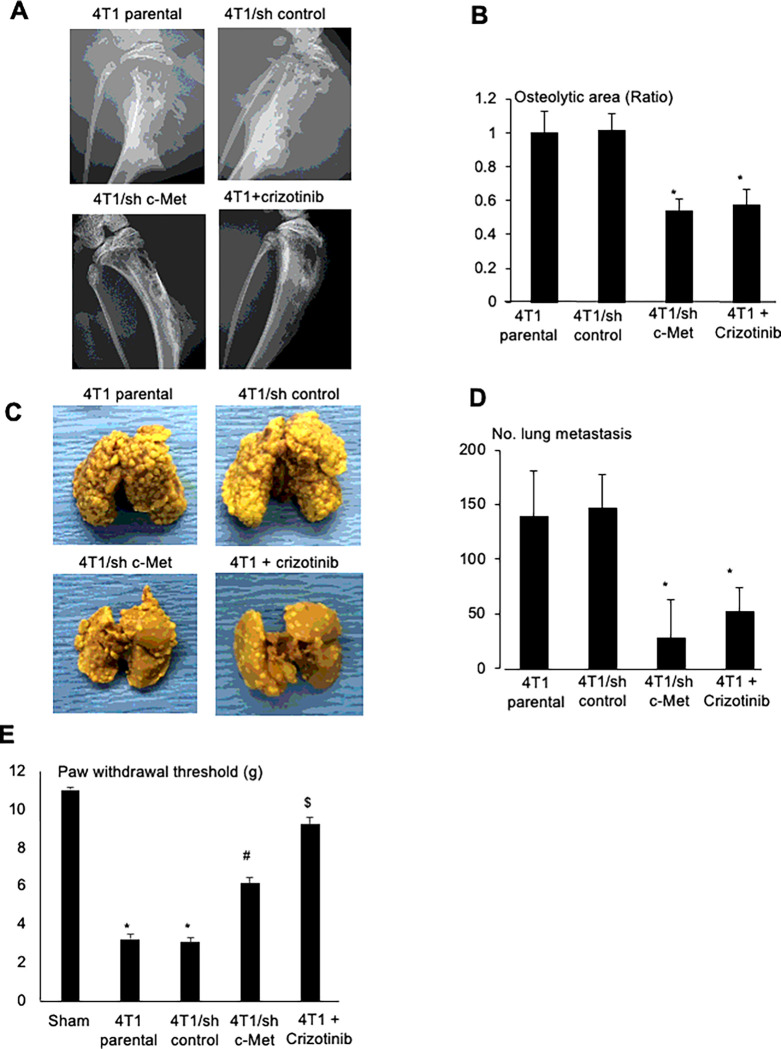
Role of HGF/c-Met in 4T1 BC progression in tibiae, lung metastasis from tibiae, and BCIBP induction in 4T1 BC mice. **A.** Effects of shRNA knockdown of c-Met in 4T1 BC cells or inhibition of c-Met signaling by crizotinib (30mg/kg, po, once every day from day 6 to 20, #4368, Tocris) on the development of osteolytic lesions associated with 4T1 BC progression in tibiae. Radiographs were taken prior to sacrifice at day 21 under general anesthesia. **B.** Quantitative analysis of Figure 7A. Ratio represents, osteolytic area of 4T1 BC + SB366791 or 4T1/sh c-Met/osteolytic area of 4T1 parental x 1. Data are shown as mean ± SD (N=4). *p<0.05 *vs* 4T1 parental and 4T1/sh control mice. **C.** Effects of shRNA knockdown of c-Met in 4T1 BC cells or inhibition of c-Met signaling by crizotinib on lung metastases from tibiae in the same experiment as Figure 7A. **D.** Quantitative analysis of Figure 7C. Number of 4T1 BC metastatic foci in lung was macroscopically counted as described in Figure 4D. Data are shown as mean ± SD (N=4). *p<0.05 *vs* 4T1 parental and 4T1/sh control mice **E.** Effects of shRNA knockdown of c-Met in 4T1 BC cells or inhibition of c-Met signaling by crizotinib on BCIBP induction assessed by hind-paw mechanical allodynia at day 21. See Figure 2A legend for experimental details. Data are shown as mean ± SD (N=4). *p<0.01 *vs* sham mice. ^#^p<0.05 *vs* 4T1 parental and 4T1/sh control mice. ^&^p<0.01 *vs* untreated 4T1 parental mice.

To validate the role of HGF in 4T1 BC progression in bone and metastasis to lung in the 4T1 BC mice, we determined the effects of crizotinib, a small compound of c-Met/ALK tyrosine kinase inhibitor, which has been shown to have beneficial effects in patients with non-small cell lung cancer ^43^ and breast cancer ^44^. Our results demonstrated that oral administration of crizotinib significantly inhibited the development of osteolytic lesions associated with 4T1 BC colonization in tibiae (Fig. 7A, bottom, right and Fig. 7B), decreased lung metastasis from tibiae (Fig. 7C, bottom, right and 7D). These results suggest that HGF promotes 4T1 BC progression in bone and associated osteolysis, and 4T1 BC metastasis to lung. Related to the promotion of lung metastasis, the DRG SN CM stimulated 4T1 BC cell migration in the wound healing assay, which was blocked by crizotinib (Supplementary Fig. 6A). Since HGF is a scatter factor ^45^, these results suggest that HGF produced by DRG SNs promotes cell migration of 4T1 BC cells, thereby increasing lung metastasis from bone. Further, mechanical allodynia was also significantly ameliorated in the 4T1 BC mice treated with crizotinib (Fig. 7E), suggesting that HGF is involved in the induction of BCIBP.

However, since crizotinib was systemically administered in the 4T1 mice, these results do not show if HGF derived from SNs innervating bone is specifically responsible. To address this, we established 4T1 BC cells in which c-Met was stably silenced by specific shRNA for c-Met (4T1/sh c-Met) and 4T1 BC cells in which scrambled shRNA was infected (4T1/sh control) as control. Transduction of these shRNAs did not change basal cell proliferation of these cells (Supplementary Fig. 5B). However, promotion of cell proliferation (Supplementary Fig. 5B) and tyrosine phosphorylation of c-Met (Supplementary Fig. 5C) by HGF was markedly decreased in 4T1/sh c-Met BC cells compared to 4T1/sh control BC cells. These results show that the responsiveness of 4T1/sh c-Met cells to HGF is significantly diminished.

We then injected parental 4T1, 4T1/sh control and 4T1/sh c-Met BC cells into the bone marrow cavity of tibiae in mice. Radiographs showed that 4T1 parental BC and 4T1/sh control cells progressively developed multiple osteolytic lesions (Fig. 7A, top and Fig. 7B). In contrast, osteolytic lesions were significantly decreased in tibiae injected with 4T1/sh c-Met BC cells (Fig. 7A, bottom, left and Fig. 7B) compared to tibiae injected with 4T1 parental or 4T1/sh control BC cells. Lung metastasis from tibiae was significantly decreased in 4T1/sh c-Met BC cells as well (Fig. 7C, bottom, left and 7D). These results suggest that HGF locally produced in SNs in bone is responsible for the promotion of 4T1 progression in bone and metastasis to lung from bone.

BCIBP was also significantly reduced in mice injected with 4T1/sh c-Met BC cells mice treated with crizotinib (Fig. 7E), suggesting that HGF plays a role in BCIBP induction.

As shown in supplementary Fig. 3, mice injected with 4T1 parental BC cells showed weight loss (Supplementary Fig. 5D). In contrast, mice injected with 4T1/sh c-Met BC cells did not lose weight. Further, treatment of 4T1 BC mice with crizotinib (Supplementary Fig. 5D, black arrows) prevented weight loss. These results suggest that HGF secreted from SNs is involved in the development of cancer cachexia presumably via modulating BC progression.

## DISCUSSION

Bone pain coupled with pathological innervation and sprouting of SNs in the tumor is one of the most common and detrimental complications of BC bone metastasis ^7–9^. Shorter survival of BC patients with bone pain than those without bone pain ^46^ led us to hypothesize that bone pain may affect BC progression in bone. To address this, we established an animal model in which inoculation of mouse 4T1 BC and E771 cells in tibiae developed an acidic microenvironment, promoted innervation and sprouting of SNs, and increased excitation of SNs following up-regulation of the expression of the acid-sensing nociceptor TRPV1, leading to the induction of BCIBP. Our results demonstrated that pharmacological blockade of TRPV1 actions using synthetic and natural antagonist and genetic ablation of TRPV1 not only inhibited SN excitation and BCIBP induction but also decreased BC progression in tibiae, and lung metastasis from tibiae. The synthetic and natural TRPV1 antagonists showed no growth-inhibitory effect on 4T1 BC cells. While TRPV1 is found to be expressed in some BCs ^33^, our data show 4T1 BC cells express little TRPV1 compared to SNs, suggesting that these antagonists show their actions through SNs rather than 4T1 BC cells. These results support our hypothesis that the TRPV1 of SNs plays important roles in the regulation of BC progression and metastasis. In agreement with our results, pharmacological ablation of the SN TRPV1 has been reported to suppress initiation and progression of pancreatic ductal carcinoma in mice ^47^. Further, consistent with previous reports ^28,29,31^, we verified that the SN TRPV1 contributes to the induction of BCIBP. Taken together, it is suggested that blocking the activation of the SN TRPV1 has anti-cancer and analgesic effects in the acidic bone microenvironment associated with BC colonization, Thus, SN TRPV1 may play as a mediator of biological interactions between BCIBP and BC progression in bone. The mechanism of shorter survival in cancer patients with pain than those without pain ^46^ remains unclear. It has been proposed that distress, anxiety, depression, uncertainty and hopelessness associated with devastating pain impact overall survival of cancer patients ^46^. Our results that the SN TRPV1 contributes to BC progression and metastasis, as well as BCIBP induction, may provide a molecular basis for the poor outcome in cancer patients with pain.

We then determined the mechanism underlying SN TRPV1 regulation of BC progression and metastasis. Previous studies described that neurotropic factors, growth factors, axonal guidance molecules and colony-stimulating factors produced in the tumor microenvironment mediate the interactions between neurons and cancer cells ^39^. We found that excited DRG SNs release increased levels of HGF, a well-known potent growth and scatter factor for BC ^41^, following activation of TRPV1 in the acidic 4T1 BC microenvironment. HGF has been known to be expressed in SNs and promote the development, differentiation, regeneration, and survival of neurons in an autocrine manner ^48^. Our data demonstrate that HGF released from excited SNs regulates neighboring BC cell progression and metastasis in bone. Taken together, we propose that increased expression of HGF in SNs following TRPV1 activation plays a role as a paracrine coordinator of the crosstalk between SNs and BC in the acidic tumor microenvironment in bone (Supplementary Fig. 6). HGF is a newly-identified product of SNs that regulates BC progression in bone.

BC in bone metastasis frequently disseminates to distant visceral organs after a persistent period of dormancy, resulting in secondary metastasis that further increases the mortality of BC patients ^17–19^. However, the mechanism of secondary visceral metastasis from bone remains poorly understood. Our results suggest that TRPV1 of SNs activated in response to the acidic BC microenvironment regulates lung metastasis from bone by increasing the expression of HGF, a potent stimulator of epithelial-mesenchymal transition and cell migration ^49^, in SNs innervating bone. Thus, bone may serve as a soil that fosters metastatic BC cells to spread to the next distant visceral organs.

Reduction of BCIBP by a c-Met inhibitor crizotinib suggests that HGF plays a role in the induction of BCIBP. Consistent with this notion, a recent clinical study reported that cabozantinib, a new generation of c-Met inhibitor, reduces bone pain in patients with castration-resistant prostate cancer ^50^. However, these agents inhibit the growth of tumor that is the primary cause of pain. It is thus unclear whether these inhibitors indirectly ameliorate BCIBP through suppression of tumor growth or direct inhibition of nociceptor activation at present.

Bone is innervated by both autonomic nerves (ANs) and SNs. We show here that SNs regulate BC progression in bone. Results are accumulating that persistent stress, anxiety, and depression are risk factors for cancer and associated with increased recurrence and mortality in women with BC via activation of the sympathetic nerve system ^51^. Consistent with these clinical studies, the β-blocker propranolol, which inhibits sympathetic nerve activation, decreased stress-induced bone metastasis of BC ^51^ and ANs contribute to the progression of prostate cancer ^52^ in preclinical models. Role of ANs and partnership between ANs and SNs in the regulation of BC progression in bone needs to be determined.

In conclusion, we propose that BC aggressiveness in bone is regulated by the release of a paracrine factor such as HGF from excited SNs innervating bone following TRPV1 activation in the acidic BC microenvironment. Blockade of the SN TRPV1 action may be a potential therapeutic approach for cancer progression in bone and its complications such as bone pain and secondary metastasis to visceral organs.

## METHODS

### Reagents

HGF was from R & D (Minneapolis, MN). 5’-Iodoresiniferatoxin (I-RTX), doxorubicin, crizotinib and SB366791 were from Tocris (R & D, Minneapolis, MN); rabbit monoclonal antibodies to, ERK1/2, CREB, pERK1/2, pCREB, p-Met, CAMKIIαand mouse monoclonal antibodies to c-Met, HRP-conjugated horse anti-mouse IgG and goat anti-rabbit IgG antibody were from Cell Signaling Technology (Danvers, MA); bafilomycin A1, rabbit polyclonal antibodies to TRPV1 and HGF, the goat polyclonal antibody against CGRP, and HRP-conjugated mouse monoclonal antibody against β-actin were from Abcam (Cambridge, UK); Alexa fluor 488-conjugated donkey anti-rabbit IgG and Alexa fluor 680-conjugated donkey anti-goat IgG and rhodamine red-X (RRX)-conjugated donkey anti-goat IgG antibody were from Jackson Laboratory (Bar Harbor, ME); Rhodamine phalloidin was from Life Technologies (Carlsbad, CA); and HRP-conjugated goat anti-chicken antibody was from Santa Cruz Biotechnology (Dallas, TX). Anti-a3V-ATPase antibody was a kind gift from Dr. Sun-Wada, Doshisha Women’s College, Japan.

### BC Cells

Mouse BC cells 4T1 have been maintained in our laboratory and mouse BC cells E0771 were purchased from CH3 Biosystems (#94A001, Amherst, NY). The 4T1 and E0771 cells and human BC cells MCF-7 and MDA-MB-231 were cultured in DMEM (Gibco, Carlsbad, CA) with 10% FBS, 100 U/ml penicillin, and 100 mg/ml streptomycin. All cell lines were analyzed and went through authentication by targeted genomic and RNA sequencing.

### DRG SNs

The lumbar (L3-L5) DRG SNs were isolated as described ^15^. Primary rat DRG neuronal cells (Lonza, R-DRG-505, Alpharetta, GA) were cultured in primary neuron growth medium (Lonza, #CC-4461) with 2% FBS, L-glutamine, gentamycin sulfate-amphotericin (GA-1000, Lonza, #CC-4083) and neural survival factor-1 (NSF-1, Lonza, #CC-4323) as instructed. Immortalized rat DRG neuronal cells, 50B11, were a generous gift from Dr. Hoke, Johns Hopkins University and cultured in Neurobasal medium (Gibco) supplemented with 10% FBS, 1 x B27 supplement (Gibco), 0.2% glucose and 0.5 mM glutamine (neuron growth medium) ^30^. The rat DRG/mouse neuroblastoma hybrid cells, F11, were cultured as described ^53^.

### Neurite outgrowth

Primary DRG neuronal cells (1 × 10^3^, lower chamber) were co-cultured with 4T1 BC cells (5 × 10^3^, upper chamber) in transwells (Corning, Corning, NY) for 72 h. Neurite outgrowth in lower chambers was visualized with calcein AM (1 μM, Life Technologies) and the length of outgrowing neurites was quantified under a fluorescent microscope using Neuron J.

### Microfluidic culture platforms

Neurite outgrowth from F11 cells in response to extracellular microenvironment of 4T1 BC cells was determined using microfluidic culture platforms ^54^. Suspension of F11 cells (10^5^ cells/10 μl) was introduced into the left channels of the microfluidic chambers (AXIS^™^, Millipore, Billerica, MA), and the right channels were filled with the neuron growth medium. After 12 h, 4T1 BC cells (10^6^ cells/ml) were plated into the right channels and cultured for further 48 h. Non-adhered cells and debris were then washed out, and the chambers were labeled with calcein AM (1μM) for 10min. The number of F11 cells extending neurites longer than 50 μm toward 4T1 BC cells were counted under a fluorescent microscope using Neuron J.

### Wound healing assay

Assay was performed as we described ^55^. The 4T1 BC cells were grown to confluence in 6-well tissue culture dishes, and a single scratch was made in the confluent monolayer using a sterile 200-μl pipette tip. The monolayer was washed with PBS, and then complete medium containing indicated inhibitors or vehicle was added. The number of cells that had migrated over the margins of the wound was counted after 12 h of treatment under a phase contrast microscope.

### c-Met Knockdown

4T1 cells were infected with 20 μl of control (sc-108080) or c-Met (sc-35924-V) shRNA lentiviral particles (Santa Cruz) in the presence of 5 μg/ml polybrene for 24 h, and cultured in DMEM with 5% FBS for 7 days in the presence of 4 μg/ml puromycin (Gibco) to select cells stably expressing the shRNAs.

### Microarray of DRGs

4-week-old female Spraque-Dawley (SD) rats weighting 250 to 300 g were used, respectively. The rats were maintained in plastic cages in a 12-hour light/12-hour dark cycle at a temperature of 21°C.

Rat pulmonary carcinoma cells (IP-B12) which show osteolytic tumor growth in bone marrow established by Dr Nakanishi^40^. IP-B12 (1×10^5^/10μl) were inoculated into the bone marrow cavity of the right tibiae in rat under general anesthesia. DRGs were collected from rat followed by the isolation of RNA using NucleoSpin RNA Plus after 14days from inoculation. DRGs of left tibia were used as control. Biotinylated cRNA were prepared according to the standard Affymetrix protocol from 100 ng total RNA. Following fragmentation, 12.5 ug of cRNA were hybridized for 16 hr at 45C on GeneChip Rat Genome 230 2.0 Array. GeneChips were scanned using the GeneChip Scanner 3000 7G. The data were analyzed with Microarray Suite version 5.0 (MAS 5.0) using Affymetrix default analysis settings.

### Intratibial injection

Mouse 4T1 or E0771 BC cells (2×10^5^/10μl) or PBS (sham) were inoculated into the bone marrow cavity of the right tibiae in 4- to 6-week-old female BALB/c (Harlan Laboratories, Indianapolis, IN), or C57BL/6J (E0771 BC) (Jackson Laboratory, Bar Harbor, ME), respectively, under general anesthesia with ketamine (Ketaset; 90–150 mg/kg, ip) and xylazine (AnaSed; 5–10 mg/kg, ip). In some experiments, mice were treated with or without antagonists, inhibitors and anti-cancer drugs as indicated in the figure legends.

### Evaluation of BCIBP

BCIBP was evaluated by hind-paw mechanical allodynia of 4T1 BC mice by von Frey test using the Dynamic Plantar Aesthesiometer (Ugo Basile, Gemonio, VA, Italy), as we described ^11,15^. The test determines the force by which rodents withdraw their hind-paw following poking by a single von Frey filament in response to automated increasing force. Rodents with pain exhibit paw withdrawal with lighter force than those without pain.

### Immunoblotting

Cells or tissues were lysed in lysis buffer (Invitrogen, Carlsbad, CA), electrophoresed on a 10% SDS-PAGE, and blotted onto PVDF membranes (Bio-Rad Laboratories, Hercules, CA). After blocking, the membranes were incubated with primary antibodies to HGF (1:1,000, Abcam, #ab83760), pc-Met (1:1,000, Cell Signaling, #3077), pERK1/2 (1:1,000, Cell Signaling, #4370), or pCREB (1:1,000, Cell Signaling, #9198) overnight at 4°C, and then with horse radish peroxidase (HRP)-conjugated secondary antibodies for 1h. Protein bands were visualized with a SuperSignal West Dura Chemiluminescent Substrate (Thermo Fisher Scientific, Carlsbad, CA) ^15^.

### Histology and immunohistochemistry (IHC)

Bones were fixed in 10% neutral buffered formalin for 48 h, decalcified in 10% EDTA for two weeks and stained with hematoxylin and eosin (HE). Lungs were fixed with Bouin’s solution and the number of metastatic foci was macroscopically counted.

For IHC, bones were fixed, decalcified, embedded in OCT compound and sectioned at 10μm thickness using a cryostat. After blocking, sections were incubated with the primary antibodies overnight at 4°C, and secondary antibodies for 60 min, mounted with coverslips in VECTASHIELD anti-fade mounting medium (Vector Laboraories Inc, Burlingame, CA) and observed under TCS SP8 confocal laser scanning microscope (Leica Microsystems, Nussloch, Germany)

Tumors and DRGs were sectioned at 10μm thickness, and incubated with primary antibodies to CD-31 (1:50, Abcam, #ab28364), DeadEnd^™^ Colorimetric TUNEL System (Promega, #G7360, Madison, WI), Ki67 (1:400, Cell Signaling, #9129), PGP9.5 (1:200, Abcam, #ab8189), CGRP (1:200 Abcam, #ab36001), TRPV1 (1:1,000 Abcam, #ab31895), peripherin (1:1,000 Abcam, #ab4666), or HGF (1:200, Abcam, #ab83760) overnight at 4°C, and a secondly fluorescent-labeled antibody (1:100) for 60 min or a streptavidin-biotin complex, EnVision HRP (Dako, Carpinteria, CA), for 60 min and visualized using a 3,3-diaminobenzidine (DAB) substrate-chromogen solution (Dako Cytomation Liquid DAB Substrate Chromogen System).

### Statistics

Data were analyzed using a Mann-Whitney or an unpaired Student’s t test for comparisons of two groups. A one- or two-way analysis of variance (ANOVA), respectively, and a Dunn’s, Dunnet’s and Tukey’s test for the analysis of multiple group comparison, using PRISM statistical software (ver. 7.0). Results are expressed as the mean ± standard deviation (SD). P < 0.05 was considered significant.

### Study Approval

All animal studies were approved by the Institutional Animal Care and Use Committee at Indiana University School of Medicine (Protocol #: 10553) and conducted according to. All study methods were performed in accordance with the ARRIVE guidelines, American Veterinary Medical Association Guidelines and regulations of this organization as well as the Ethical Guidelines for Medical and Health Research Involving Human Subjects in Japan.

## Data Availability

The datasets generated and analysed with microarray are available in the GEO repository, [GSE234650 study at: https:/www.ncbi.nlm.nih.gov/geo/query/acc.cgi?acc=GSE234650
